# Circularly Polarized Stimulated Emission from a Chiral Cavity Based on Apparent Circular Dichroism Organic Thin Films

**DOI:** 10.1021/acsphotonics.4c02560

**Published:** 2025-03-19

**Authors:** Li-Zhi Lin, Ling-Qi Huang, Shi-Wei You, Yi-Jan Huang, Francesco Zinna, Andrew Salij, Lorenzo Di Bari, Randall H. Goldsmith, Roel Tempelaar, Chia-Yen Huang, Tzu-Ling Chen

**Affiliations:** † Department of Photonics, College of Electrical and Computer Engineering, 34914 National Yang Ming Chiao Tung University, No. 1001, Daxue Road, Hsinchu City 300093, Taiwan; ‡ Dipartimento di Chimica e Chimica Industriale, 9310 Università di Pisa, Via Giuseppe Moruzzi, 13, Pisa, Pisa 56124, Italy; § Department of Chemistry, 3270 Northwestern University, 2145 Sheridan Rd, Evanston, Illinois 60208, United States; ∥ Department of Chemistry, University of Wisconsin-Madison, 1101 University Ave, Madison, Wisconsin 53706, United States; ⊥ Theoretical Division, Los Alamos National Laboratory, Los Alamos, New Mexico 87545, United States

**Keywords:** organic thin films, circular polarization, dye laser, apparent circular dichroism (ACD), chiral laser, stimulated emission, degree of circular polarization, dissymmetry factor

## Abstract

The lack of intrinsic mirror symmetry in cavity mirrors poses a significant challenge for most organic chiral materials in generating circularly polarized (CP) lasers. However, nonreciprocal chiroptical materials, such as recently developed organic thin films exhibiting apparent circular dichroism (ACD), provide a promising approach to CP light generation. In this work, we integrate an ACD-based thin film into a free-space dye laser cavity, achieving direct CP laser emission with a degree of circular polarization (DOCP) up to 0.6, corresponding to a dissymmetry factor (*g*_lum_) of 1.2, a new record for organic chiral lasers. The degree of polarization (DOP) is close to 0.8, and the observed ellipticity in the emitted light originates from the ACD effect in the thin film, leading to asymmetric cavity losses for right- and left-circularly polarized light. This breakthrough demonstrates the potential of ACD-based materials to overcome the limitations of conventional chiral laser systems, marking a significant advancement in the field and paving the way for next-generation chiral photonic devices.

## Graphical abstract

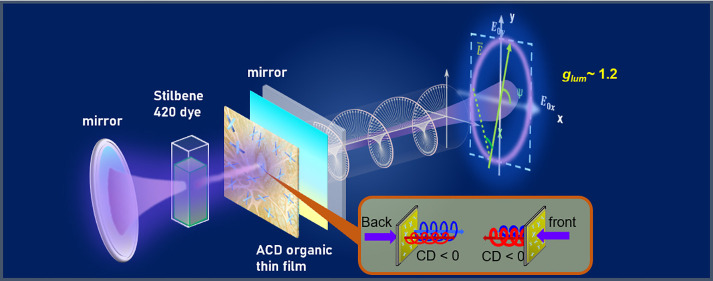

## Introduction

Circularly polarized (CP) light has garnered significant attention due to its extensive applications across fields such as optoelectronic devices, spintronics, optical communication of spin information, and optical sensing and imaging.^1–5^ The ability to generate and control CP light is essential, as the handedness—left- (LCP) or right-circularly polarized (RCP)—plays a distinct role in modulating light-matter interactions. Effective control over CP light is crucial for advancing technologies that rely on polarization-sensitive processes, such as chiral recognition and quantum information processing.^6^

The advent of metamaterials has advanced CP light generation by enabling precise control over polarization and light propagation.^7^ These structures manipulate light on subwavelength scales to achieve tailored optical properties. While metasurfaces offer promising polarization control, they often require complex fabrication and hybridization with optically active materials^8^ for CP emission in laser systems. In contrast, organic chiral materials provide a simpler and more adaptable alternative,^9,10^ facilitating direct chiral light-matter interactions.^11–13^ Integrating organic chiral thin films into laser cavities presents a flexible, cost-effective method for generating CP light, with enhanced efficiencies, tunability, and a promising pathway toward chiral optoelectronic devices and polariton lasers.^14,15^

Organic compounds composed of chiral molecules exhibit intrinsic photophysical properties such as circular dichroism (CD) and circularly polarized luminescence (CPL), which are the differential absorption and emission of left- and right-handed light.^16^ Despite their potential for generating CP light, most small molecules exhibit weak CD, leading to low dissymmetry factors (*g*_lum_ < 10^–3^) in the visible range.^17^ While larger dissymmetry factors (greater than 0.2) can be achieved through advanced fabrication methods,^13,18,19^ these systems very rarely produce a coherent and highly polarized luminescence which would be necessary for stimulated CP-emission. The prospect of coherent CP laser light, which can reach intensities over 10^8^ times greater than that of fluorescence, remains particularly compelling.

Systems utilizing chiral nematic liquid crystals,^20,21^ nanocrystal cavities,^22^ low-symmetry 2D materials,^23^ or optical retardation techniques^24^ have been extensively studied for CP laser generation. Achieving organic CP lasers generally requires a laser gain medium combined with a chiral environment. However, typical optical cavities do not enhance chiroptical activity due to the lack of intrinsic mirror symmetry^25^ and the reciprocal optical properties of most chiral materials, which do not vary with sample orientation.^26^ Attempts at direct CP lasing, such as using chiral BODIPY-type laser dyes^27^ or chiral biomolecules,^28^ have yielded dissymmetry factors up to 0.29. Nonetheless, these limitations highlight the need for further advancements in the CP laser technology.

Our work introduces a novel approach to generating CP light by incorporating a unique chiral organic thin film with nonreciprocal CD properties.^29,30^ This nonreciprocal behavior, known as apparent circular dichroism (ACD), originates from the material’s 2D chirality of oriented molecules,^31,32^ which results in directional-dependent CD. The film displays nearly opposite CD responses when illuminated from its front and back surfaces, leading to an accumulated asymmetry in the absorption of RCP and LCP light over multiple passes within the cavity.^33^ This induced asymmetry in cavity loss has the potential to significantly enhance the degree of circular polarization (DOCP) in the laser output.

To address this, we designed a free-space dye laser system with one of the cavity mirrors coated with a thin film made of phenylene bis-thiophenylpropynone (PTPO, see Figure 2). PTPO thin films have shown efficient nonreciprocal CP properties both in absorption and emission.^34^ The aim was to investigate whether the film’s enhanced asymmetric absorption of RCP and LCP light could lead to the generation of CP laser emission. In our experiments, we achieved a DOCP of up to 0.6—substantially higher than previously reported for organic CP lasers. This result not only demonstrates the ability of self-assembled chiral films to overcome the limitations of traditional chiral media but also provides a novel approach for generating strong CP emission through cavity engineering. Our findings open new avenues for advanced photonic devices and offer deeper insights into chiral light-matter interactions in stimulated emission processes.

## Experimental Setup

### ACD Organic Thin Film

The ACD organic thin film is obtained through the self-assembly of organic π-conjugated dyes into chiral aggregated supramolecular structures, enhancing the intrinsic 2D chirality and exhibiting nonreciprocal chiroptical properties due to the ACD effect.^32,35^ The total CD of this material is described by

$${\mathrm{CD}}_{\mathrm{abs}}={\mathrm{CD}}_{\mathrm{iso}}+\frac{{\mathrm{LD}}^{\prime }\cdot \mathrm{LB}-\mathrm{LD}\cdot {\mathrm{LB}}^{\prime }}{2}$$ 1

The first term represents the intrinsic isotropic component, corresponding to reciprocal light-matter interactions. The second term, referred to as ACD,^31,36^ accounts for the interference between linear dichroism (LD) and linear birefringence (LB), with LD’ and LB’ referring to the 45-degree orientations relative to a chosen axis of LD and LB, respectively, and for this reason, it is also referred to as the LDLB effect.^29^ This nonreciprocal term arises from the preferential orientation of mesoscopic domains and is influenced by the material’s directional anisotropy and how the material is processed.^30^

The material used in this work is based on a chiral phenylene bisthiophenylpropynone (PTPO) derivative, whose films, spin-coated from CH_2_Cl_2_ solutions and thermally annealed,^34^ displayed nearly mirror-image CD spectra when illuminated from the front or back, as shown in the Figure 1a, where the CD ranges from 0.02 to 0.06 (Δ*A*) across the laser emission regime, corresponding to a dissymmetry *g*-factor (*g*_abs_ = CD/Absorbance) of around 0.07 to 0.2. The thin films had a thickness of approximately 300 nm.

**Figure 1:**
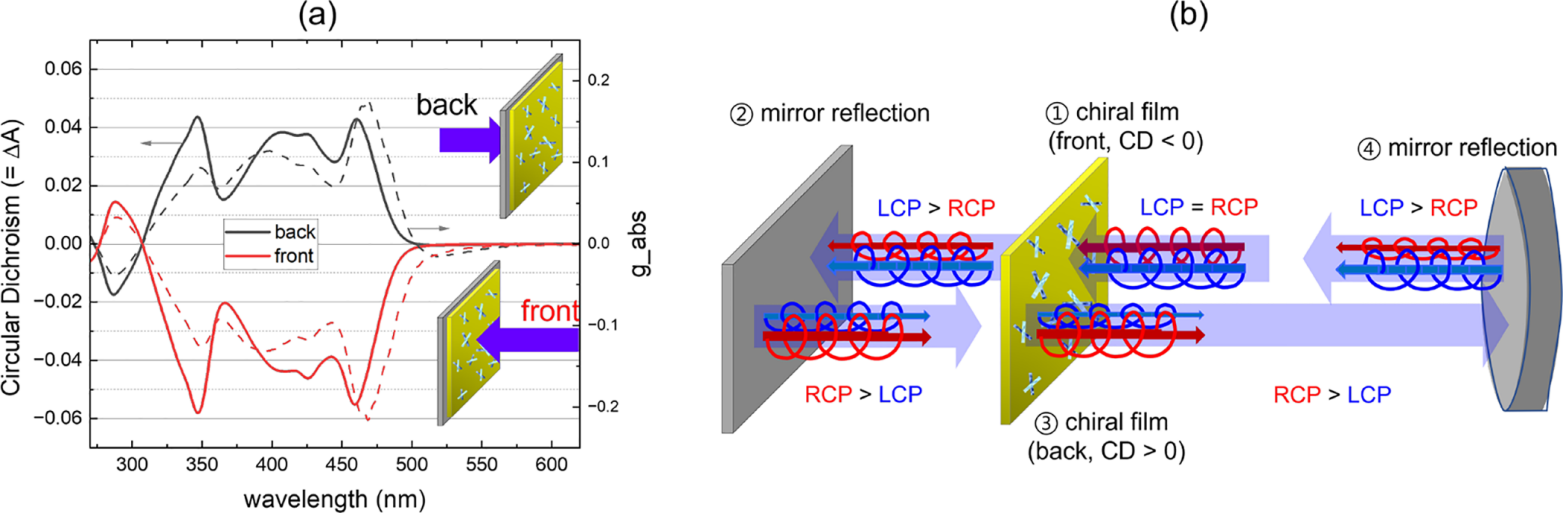
(a) CD spectrum of the PTPO organic thin film (thickness = 300 nm), exhibiting its nonreciprocal chiroptical behavior. The nearly mirror-image CD spectra upon flipping the sample highlight the directional dependence of the film’s CD properties. (b) Schematic representation of the nonreciprocal chiroptical effect of the PTPO thin film within the laser cavity (excluding the laser gain medium, which initially generates linearly polarized light, resulting in equal photon number of RCP and LCP). The scheme illustrates how the PTPO thin film differentially absorbs RCP and LCP light throughout a complete cavity round trip, highlighting the asymmetric absorption behavior. This differential absorption leads to asymmetric cavity losses, effectively enhancing the CD and inducing a preferential CP state in the emitted laser light.

Earlier research has shown that these chiral films can enhance CD effects in passive optical cavities,^33^ significantly influencing the balance of cavity transmission between RCP and LCP light. However, these works have not explored whether such films can actively generate CP light when coupled with a laser gain medium. The concept of a 2D chiral cavity incorporating a nonreciprocal 2D chiral material is illustrated in Figure 1b. In this configuration, the cavity is formed by a flat mirror coated with a PTPO thin film and a concave standard mirror, which together provide the optical feedback required for lasing. Assuming the initial light is linearly polarized (comprising equal intensities of RCP and LCP light), it encounters the 2D chiral film where the CD is negative, causing greater absorption loss for RCP compared to LCP. In step 2, when this CP light reflects off a standard mirror at normal incidence, the handedness reverses—RCP becomes LCP, and vice versa. Upon re-entering the chiral film from the opposite side in step 3, where the CD is positive, LCP now experiences more absorption loss than RCP. In step 4, the mirror again reverses the handedness. Thus, within each round trip, the chiral film’s directional dependence causes alternating asymmetrical losses for RCP and LCP, shifting the balance between them and creating a cumulative asymmetry in cavity losses.

### Laser Design and Configuration

Figure 2 illustrates the concept of the laser design and the experimental setup used for spectroscopic and polarization characterization. The pump source for the dye laser is a third harmonic generation from a pulsed optical parametric oscillator (OPO) laser (Amplitude-Laser, Surelite Ex), driven by a pulsed Nd:YAG laser. The pump laser operates at 355 nm with a pulse width of 3–5 ns, a repetition rate of 10 Hz, and a polarization extinction ratio exceeding 1 × 10^–3^. A half-wave plate (HWP), positioned after the attenuator, is used to control the polarization direction of the pump laser, enabling precise tuning between transverse electric (TE) and transverse magnetic (TM) polarization modes during dye excitation.

**Figure 2:**
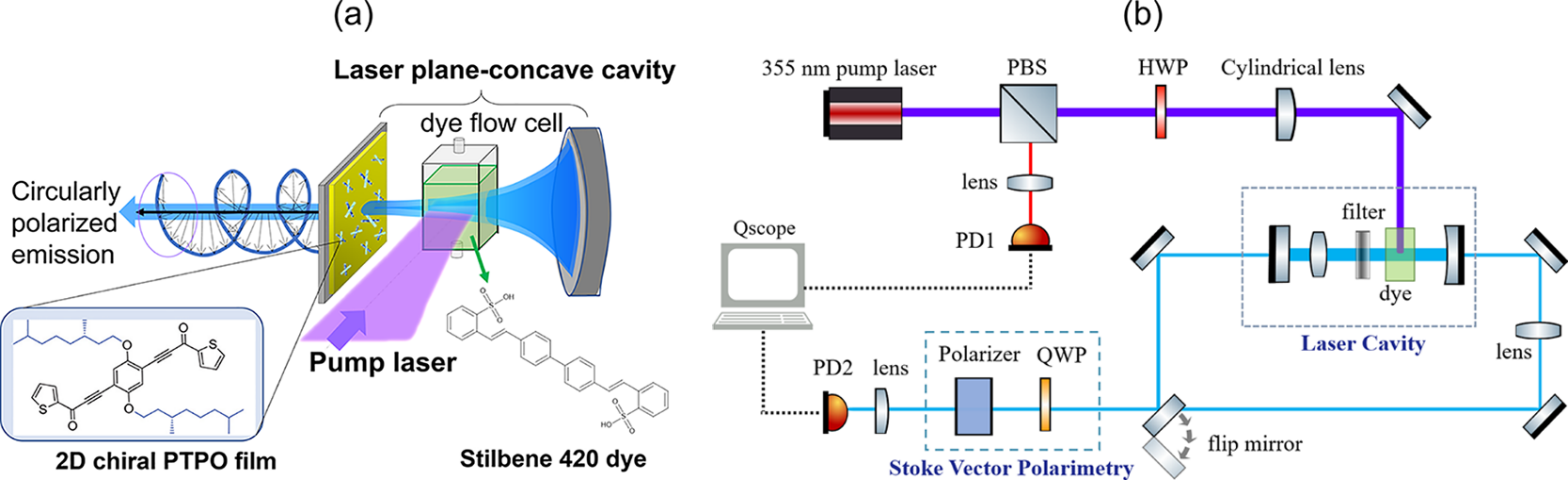
(a) Conceptual illustration of the laser cavity and pump laser configuration showing the PTPO thin-film-coated mirror and the resulting laser emission, along with its polarization ellipse. The inset displays the chemical structure of the PTPO derivative used to fabricate the thin film. (b) Experimental setup for characterizing both the laser emission spectrum and the polarization state. The 355 nm pump pulsed laser source, and the output from the dye laser cavity are monitored by photodetectors (PD1 and PD2, respectively) and a Stokes vector polarimetry system. The pump laser polarization is controlled using a half-wave plate (HWP), and the laser cavity contains a lens, a filter, and liquid dye, Stilbene 420, as the gain medium. Data acquisition is performed using an FPGA-based Qscope board.

The optical cavity features a plano-concave design, with the concave mirror reflection of 99%, and the planar mirror, coated with a high-reflectivity (HR) coating (*R* = 95%, 420–650 nm), as well as the chiral PTPO thin film. The cylindrical lens focuses the pump beam into a narrow line with a beam size of about 0.1 mm × 10 mm to maximize the overlap between the pump and dye laser cavity resonant modes. We used a 0.22 g/L solution of Stilbene 420 dye^37^ in an ethanol–water mixture (2:1 ratio). To mitigate issues associated with long-lived triplet states and thermal effects (heat accumulation), which can degrade performance in most liquid dye laser systems,^38^ we implemented a custom-built dye circulation system. In this system, the dye solution is pumped through a cuvette (1 × 1 × 10 mm^3^) with polished sides.

Due to substantial peak-to-peak fluctuations (greater than 30%) in the pump laser pulse energy, two photodetectors (PD1 and PD2) were used to simultaneously record the pulse energies of both the pump laser and the dye laser. PD1 monitored the pump pulse energy to enable normalization of the dye pulse energy, which was measured by PD2. Data acquisition was conducted by using a Field Programmable Gate Array (FPGA)-based Qscope board operating at 250 MS/s.

### Characterization of Laser Polarization States

Given that the polarization of the dye laser in the chiral cavity configuration is expected to exhibit partial polarization and ellipticity rather than perfect circular polarization, we employ the global polarization state description using Stokes parameters, which can provide the degree of polarization (DOP), orientation, and ellipticity of the polarization ellipse.

Most commercial polarimeters are not suitable for low repetition frequency light sources, so we employed a rotating quarter-wave plate (QWP) measurement system^39^ as our home-built polarimeter. The reliability of the home-built polarimeter was verified by comparing its results with those obtained from a commercial instrument (Thorlabs PAX1000VIS) using a continuous-wave (CW) light source, with further details provided in the Supporting Information. This setup includes a rotating QWP and a linear polarizer, as depicted in Figure 2a, to measure the Stokes vector of the output dye laser emission. The discrete intensities of the optical beam measured at different rotating angles of the QWP are given by

$$I\left(\theta_{n}\right)=\frac{1}{2}\left(A+B\sin\;2\theta_{n}+C\cos\;4\theta_{n}+D\sin\;4\theta_{n}\right)$$ 2

where θ_*n*_ denotes the angle of the QWP, and *A*, *B*, *C*, and *D* are constants that describe the polarization state. According to Nyquist’s sampling theorem, the maximum frequency corresponds to 4θ, so determining *A*, *B*, *C*, and *D* requires a minimum of 8 measurements of different angles.

The parameters *A*, *B*, *C*, and *D* are calculated from the intensity measurements at different angles using the following expressions:

$$\begin{array}{ll} \begin{array}{cc} A & =\frac{2}{N}\sum_{n=1}^{N}I_{n}, \end{array} & \begin{array}{cc} B & =\frac{4}{N}\sum_{n=1}^{N}I_{n}\sin\;2\theta_{n}, \end{array}\\ \begin{array}{cc} C & =\frac{4}{N}\sum_{n=1}^{N}I_{n}\cos\;4\theta_{n}, \end{array} & \begin{array}{cc} D & =\frac{4}{N}\sum_{n=1}^{N}I_{n}\sin\;4\theta_{n} \end{array} \end{array}$$ 3

where *N* denotes the total number of measured angles. In this work, the angles used are 0°, 22.5°, 45°, 67.5°, 90°, 112.5°, 135°, and 157.5°, with an additional 180° to verify the consistency when the QWP returns to the 0° position.

The Stokes parameters *S*_0_, *S*_1_, *S*_2_, and *S*_3_, are then determined as

$$S_{0}=A-C,\;S_{1}=2C,\;S_{2}=2D,\;S_{3}=B$$ 4

which can be represented on the Poincaré sphere, providing a visual representation of the polarization state. Additionally, the DOP, the DOCP, the degree of linear polarization (DOLP), and the orientation and ellipticity angles, 2ψ and 2χ, respectively, can be used to characterize the extent and nature of polarization:

$$\begin{array}{rll} \mathrm{DOP} & =\frac{\sqrt{S_{1}^{2}+S_{2}^{2}+S_{3}^{2}}}{S_{0}}, & \\ \mathrm{DOLP} & =\frac{\sqrt{S_{1}^{2}+S_{2}^{2}}}{S_{0}}, & \mathrm{DOCP}=\frac{S_{3}}{S_{0}},\\ 2\psi & =\tan^{-1}\left(\frac{S_{2}}{S_{1}}\right), & 2\chi =\tan^{-1}\left(\frac{S_{3}}{\sqrt{S_{1}^{2}+S_{2}^{2}}}\right) \end{array}$$ 5

These quantities provide a comprehensive description of the polarization state of the light beam. To accurately compute the final Stokes vector, the data in Figure 3b taken at 8 discrete angles of QWP were binned to minimize noise and enhance accuracy.

**Figure 3:**
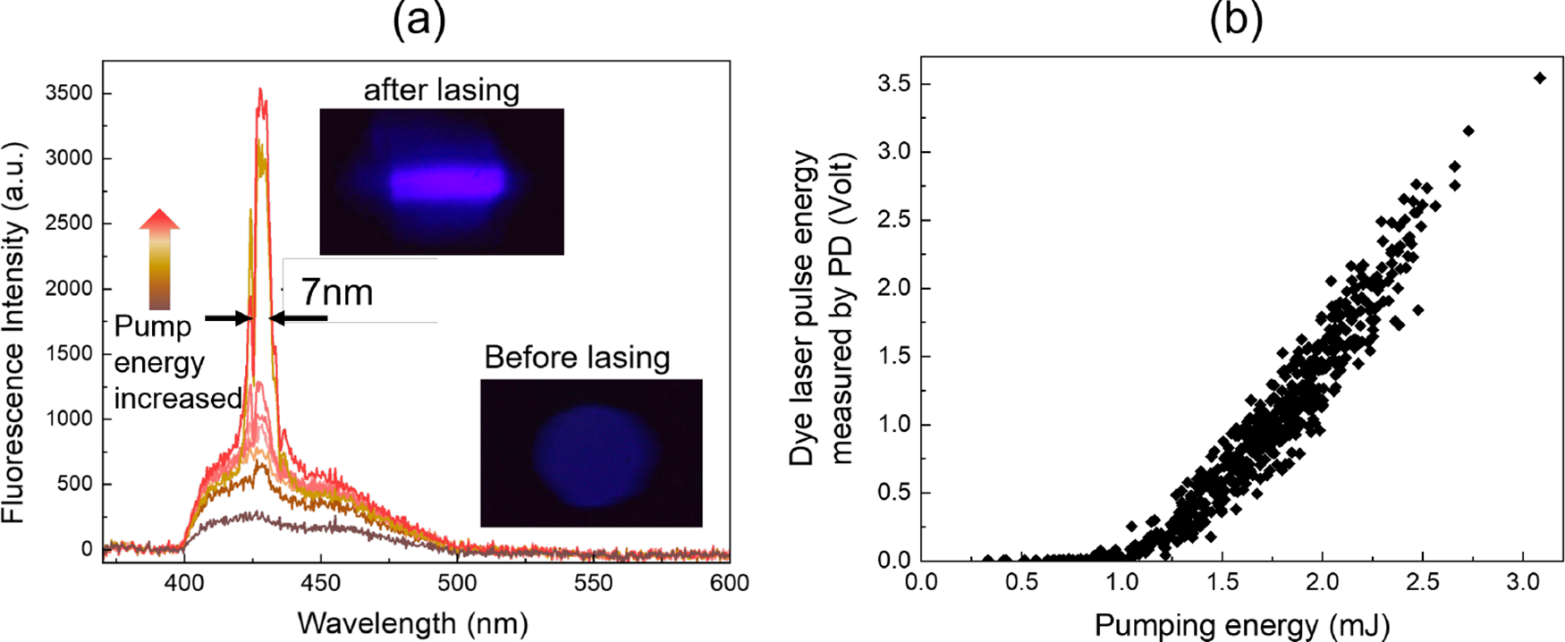
(a) Emission spectrum captured from the side view of the liquid dye cuvette with a clear reduction in full width at half-maximum (fwhm) as the averaging pump energy exceeds 2 mJ, indicating the transition from spontaneous emission to stimulated emission. The insets show two distinct beam profiles observed at the laser mirror output for different pump energies. (b) The relationship between the pump energy and the output laser energy, as detected by PD1 and PD2, reflects the lasing threshold.

## Results

### Luminescence and Laser Measurements

Figure 3a presents the emission spectra of the dye laser at different pump energy levels. Figure 3b shows the relationship between the output laser energy and the pump energy, as measured by photodetectors PD1 and PD2, with the latter calibrated with an energy meter. Below the lasing threshold (approximately 1.2 mJ), the system primarily exhibits spontaneous emission, with fluorescent photons generated by the excited dye molecules in the liquid gain medium. The cavity provides optical feedback, which is crucial for stimulating the emission of these photons and facilitating the lasing process. The resonant condition of the cavity limits the modes that can persist in the system, with longitudinal modes falling beyond the resolution of our spectrometer. Once the threshold is surpassed, a rapid transition to stimulated emission occurs, leading to a sharp increase in output power and a significant reduction in line width to around 7 nm, compared to the broader line width of the spontaneous fluorescent emission peak. This line width is consistent with those observed in other organic dye laser systems.^38,40,41^ The laser power output from the plane mirror is about five times higher than that from the concave mirror, as expected, based on the ratio of mirror reflectivities.

### Polarization States Characterization

The polarimetric analysis shown in Figure 4 presents key polarimetric parameters plotted as a function of pump energy. The total intensity, represented by *S*_0_, is directly proportional to the pump energy and exhibits a trend similar to that shown in Figure 3b. In both figures, the lasing threshold is clearly identifiable.

**Figure 4:**
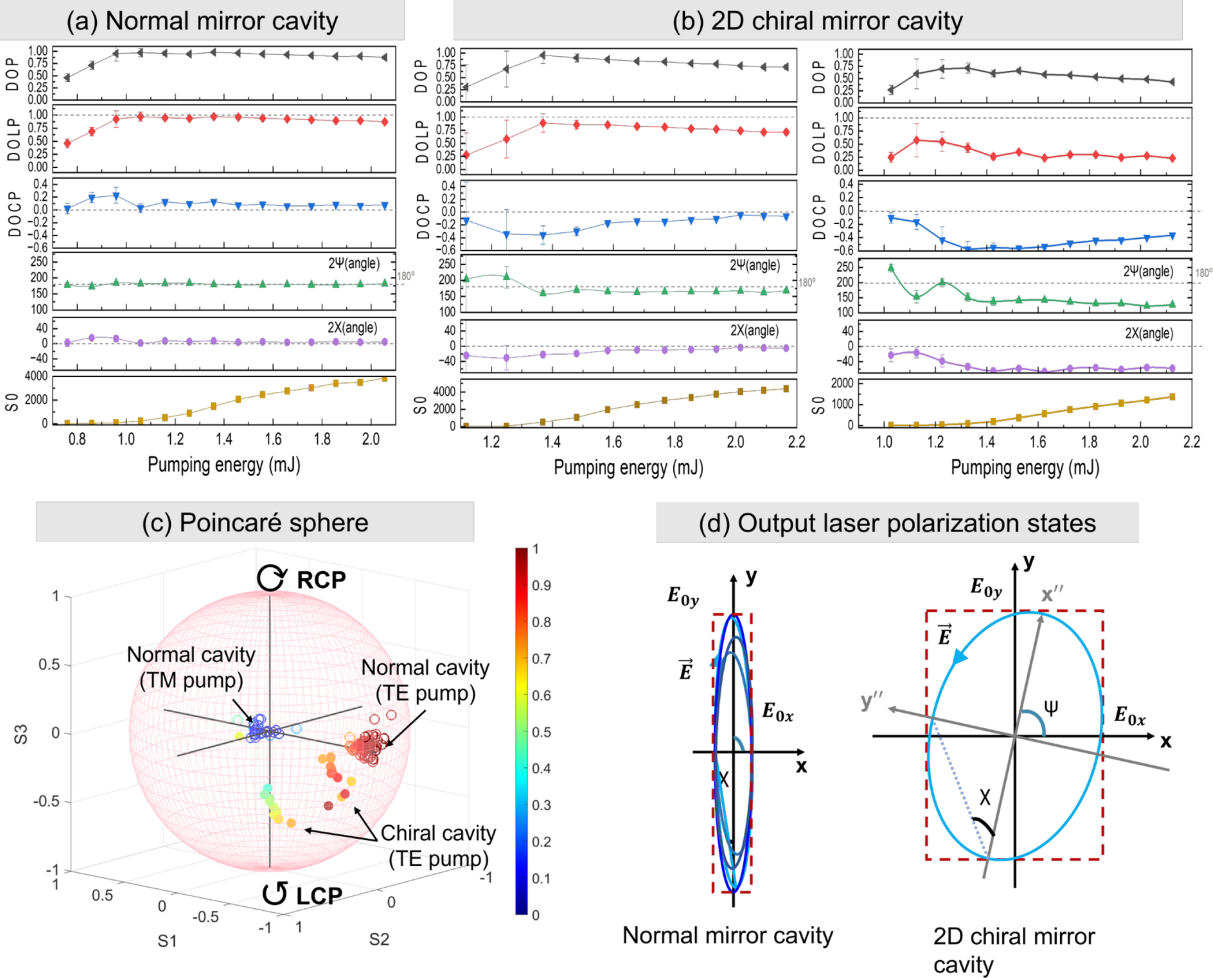
Influence of pump energy and polarization on the dye laser’s polarization state. Panels (a) and (b) display the laser emission parameters, including the orientation angle (2ψ) and ellipticity angle (2χ), as functions of pump energy for a normal mirror cavity and a 2D chiral mirror cavity, respectively. The 2D chiral mirror comprises a high-reflectivity (HR) coverslip mirror coated with a 300 nm PTPO thin film. The comparison reveals the impact of the 2D chiral film on the polarization state, particularly the enhancement of circular polarization, as indicated by changes in the 2χ. Panel (c) shows measurements from both the normal and 2D chiral mirror cavities, with measurements taken from two different lasing spots on the 2D chiral mirror. Panel (d) illustrates the elliptically polarized states of the emitted light between the normal mirror and the 2D chiral mirror cavities.

To validate the system performance, we first measured the dye laser emission in a normal mirror cavity configuration with both TE- and TM-polarized pump light. A TE-polarized pump results in a high DOLP, as shown in Figure 4c, while a TM-polarized pump leads to an unpolarized output. This behavior can be attributed to the alignment of the pump polarization with the cavity feedback direction.^42^ For TE-polarized light, the electric field aligns with the cavity polarization mode, enhancing the interaction with the dye molecules and promoting efficient lasing. In contrast, for TM-polarized light, the electric field is misaligned with the cavity mode, reducing the lasing efficiency and producing an unpolarized output. This phenomenon is consistent with previous observations in other solid-state organic and achiral dye laser systems.^40,43–45^

Figure 4a,b shows the dependence of output polarization on the pump laser polarization for a normal mirror cavity and a 2D chiral mirror cavity, respectively, with the pump being TE-polarized in both cases. For the normal cavity, the DOP increases steadily with pump energy, even below the lasing threshold, eventually reaching near-perfect linear polarization (DOP > 0.9), while the DOCP remains negligible. This indicates that the laser emission is predominantly linearly polarized along the vertical axis. The DOP shows a rapid increase near the lasing threshold, a trend similar to observations in other solid-state laser systems.^2^ In contrast, cavities equipped with PTPO-coated chiral mirrors demonstrated significantly different behavior. A significant enhancement in the DOCP up to −0.6 (*g*_lum_ = – 2 × DOCP ∼ 1.2), representative of a 60% global ellipticity, is observed near the lasing threshold. This DOCP peaked before gradually decreasing at higher pump energies.

Figure 4c shows the measured Stokes vectors for both normal and chiral cavity configurations, visualized on the Poincaré sphere with the corresponding elliptically polarized states presented in Figure 4d. The color bar represents the DOP, with variations in color corresponding to different polarization states. In the normal cavity, under both TM and TE pump configurations, the Stokes vectors cluster near the equator, indicating predominantly linear polarization with minimal circular polarization. In contrast, the chiral cavity exhibits a broader spread along the axis, particularly with the TE pump, where several points show negative values, reflecting significant circular polarization. These results demonstrate the ability of using 2D chiral PTPO to generate CP light at varying levels of DOP and DOCP, with the strongest DOCP observed in the chiral cavity.

## Discussion

To explain the trends of DOCP in Figure 4 and the influence of pump energy on the polarization state, we explore the fundamental mechanism behind the generation of the DOCP during the pulse evolution process under stimulated emission in our chiral laser system. Under vertically polarized pump light, the achiral dye laser medium primarily emits linearly polarized light (as experimentally confirmed in the normal cavity configuration), which can be treated as having equal photon populations for RCP and LCP light. The DOCP in the simulation is given by

$$\mathrm{DOCP}(t)=\frac{n_{\mathrm{RCP}}(t)-n_{\mathrm{LCP}}(t)}{n_{\mathrm{RCP}}(t)+n_{\mathrm{LCP}}(t)+n_{\mathrm{sp}}}=-\frac{g_{\mathrm{lum}}}{2}$$ 6

where *n*_RCP_(*t*) and *n*_LCP_(*t*) are the time-dependent photon populations for RCP and LCP states, respectively, and *n*_sp_ represents the background photon population, mainly contributed by spontaneous emission. In the stimulated emission regime, the lasing photon population is much greater than that of *n*_sp_. These populations are determined by the population inversion *N*(*t*) and cavity losses, with the former driven by the pump pulse energy, which we assume follows a Gaussian profile (with a pulse width of 5 ns) in the time domain. The rate equations satisfy^46^:

$$\begin{array}{r} \frac{dN(t)}{dt}=R_{p}(t)-\frac{N(t)}{\tau }-\beta \cdot c\cdot n(t)\cdot N(t)\\ \frac{dn(t)}{dt}=\beta \cdot c\cdot n(t)\cdot N(t)-\frac{n(t)}{\tau_{c}} \end{array}$$ 7

Eq 7 presents the rate equations for the time evolution of photon number density *n*(*t*) and population inversion *N*(*t*) in a laser system, including the pump rate *R*_p_, the upper state lifetime constant τ, and the stimulated emission cross-section β. The terms *N*(*t*)/τ and β · *c* · *n*(*t*) · *N*(*t*), where *c* is the speed of light, represent the spontaneous and stimulated emission terms, respectively. They describe the interaction between photon density and population inversion during the stimulation of the emission process.

Cavity losses, represented by the cavity lifetime τ_c_, is given by

$$\tau_{c}=-\frac{2L}{c}\frac{1}{\ln[R_{1}R_{2}(1-(1-e^{-A}+\delta ){)}^{2}]}$$ 8

where *L* is the cavity length, δ is other losses (such as scattering, additional absorption, etc.), *R*_1_ and *R*_2_ are the mirror reflectivities. The term 1 – *e* ^–*A*^ + δ represents the total internal loss, where 1 – *e* ^–*A*^ accounts for the absorption of RCP or LCP light induced by the PTPO thin film. The difference in A between RCP and LCP stems from the ACD of the film, leading to differing losses for RCP and LCP light in both lasing directions.

Figure 5 depicts the simulated DOCP and total photon number (proportional to *S*_0_) as functions of pump rate. The center and right panels show the variation of the peak pump rate *R*_p_ and the population inversion *N*(*t*) (upper panel) and photon population of LCP and RCP (lower panel) during the gain switching process under different pump energy. When excited by the pump laser, the population inversion, *N*(*t*) increases rapidly. This behavior is typical in processes such as gain switching, where the pump rate *R*_p_ is higher than that in steady-state lasing. We assume that in an achiral dye gain medium, which lacks intrinsic chirality, the medium itself does not preferentially interact with CP light. Therefore, the gain for both LCP and RCP light is generally the same. While the gain and standard losses remain identical for both RCP and LCP, the nonreciprocal nature of the ACD in the PTPO thin film causes an asymmetry in the absorption between these polarization states. Unlike reciprocal effects, this nonreciprocal absorption persists over successive round trips within the cavity, creating an imbalance between RCP and LCP that manifests as the observed DOCP in the emitted laser light. A similar decreasing trend in DOCP has been observed in chiral emission from biological microlasers.^28^

**Figure 5:**
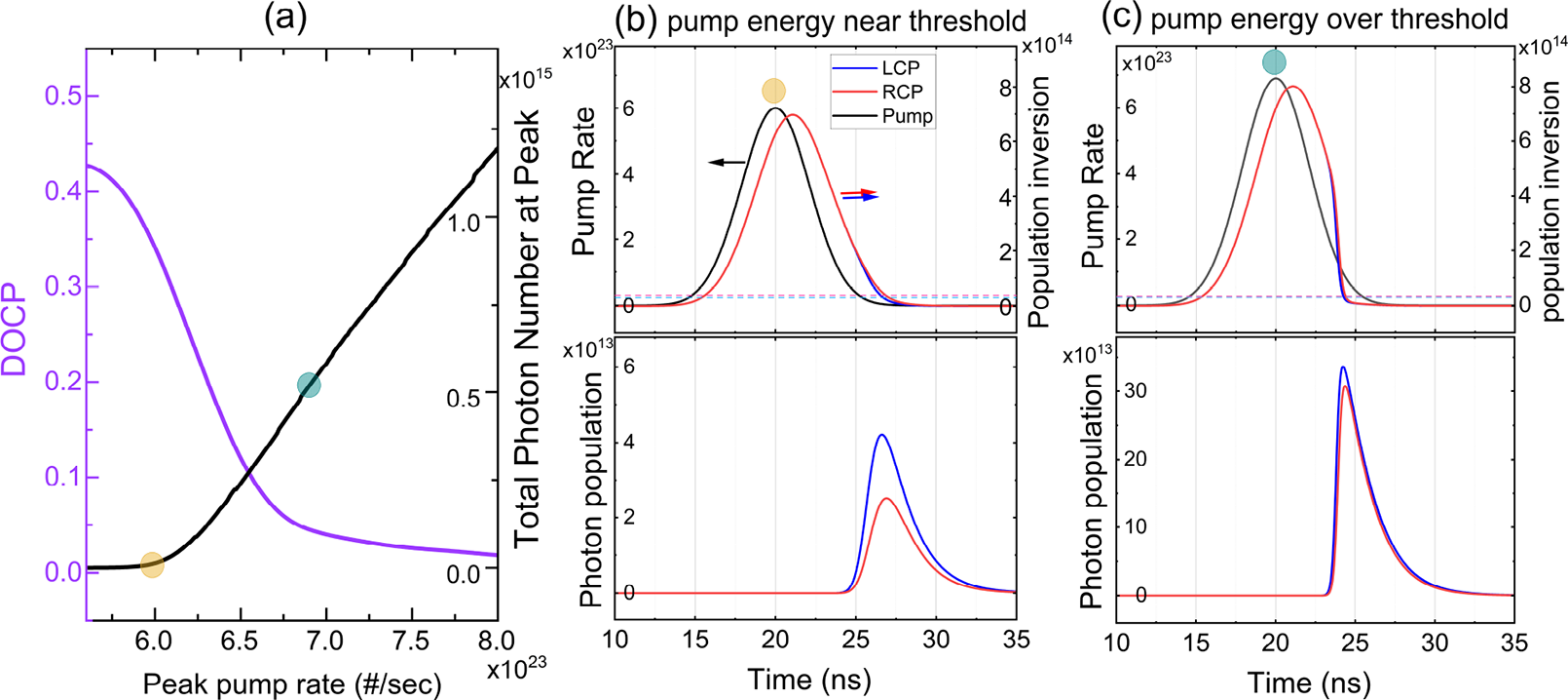
(a) Simulated degree of circular polarization (DOCP) and total photon number as functions of the pump rate (proportional to energy). (b, c) Depicts the pulse behalves under different pump energy conditions, where the dashed lines indicate the lasing threshold, determined by cavity losses. Near the threshold (b), population inversion and photon emission for LCP and RCP are nearly symmetric due to similar gain; however, differential cavity losses induced by the 2D chiral thin film introduce asymmetry, resulting in a higher DOCP. Beyond the threshold (c), increased gain reduces the influence of differential losses, leading to a decrease in DOCP. Simulation parameters: τ = 1.32 ns, β = 6× 10^–16^ cm^–2^, τ_c_ = 1.62 ns (RCP) and 1.545 ns (LCP), corresponding to an absorption A = 0.35 and absorption difference Δ*A* = 0.06, with additional internal losses δ = 0.14.

Figure 6 presents a comparison between the simulated results (purple/blue curves) and the experimental data (black points) for normalized DOCP and normalized DOCP/*S*_0_, showing excellent agreement. The fitted pump rate of 6.0 × 10^23^/s corresponds to a pump energy of 1.6 mJ, which closely matches the measured value. In the simulation, the DOCP is derived by assuming a perfect DOP of 1. For the experimental data, since the DOP is not ideal, the DOCP is normalized by the DOP for direct comparison with the simulation. Near the lasing threshold (pump energy around 1.2 mJ), this differential loss is significant, but as the pump energy continues to increase, the gain surpasses the influence of the differential loss. Consequently, the asymmetry in loss becomes less significant, causing the DOCP to decrease from its peak at higher pump energies. When DOCP is normalized by the total pulse energy and DOP (right panel), an exponential decay is observed, revealing a gradual decrease in DOCP as the pump energy increases. This suggests that the effect of differential losses between RCP and LCP photons, caused by the nonreciprocal PTPO thin film, becomes less significant at higher pump rates, where gain exceeds differential absorption.

**Figure 6:**
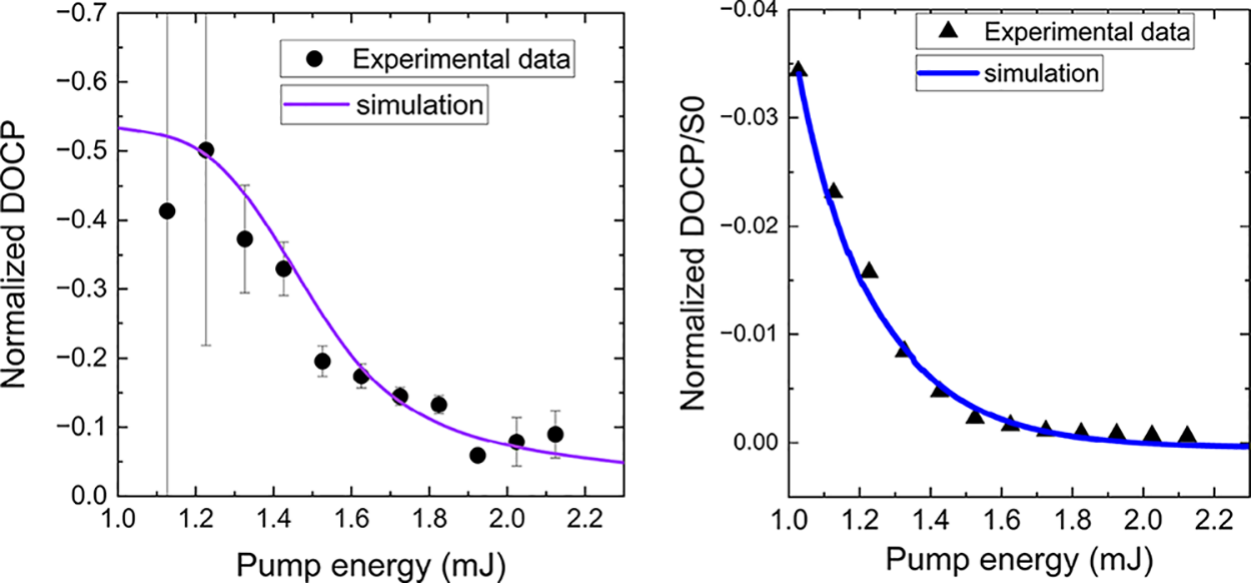
Influence of the pump rate on the polarization state of the dye laser emission. The simulation and experimental data show the evolution of DOCP (left panel) and DOCP/*S*_0_ (right panel) as a function of the pump rate, where DOCP peaks near the lasing threshold and declines at higher pump rates due to the overwhelming gain, making the asymmetric cavity loss less impactful. Simulation parameters: τ = 0.82 ns, β = 3× 10^–16^ cm^–2^, τ_c_ = 3.24 ns (RCP), and 3.09 ns (LCP).

These results highlight that the final DOCP is primarily influenced by two factors: the total number of linearly polarized photons, mainly originating from laser-induced stimulated emission in the achiral dye gain medium pumped by a linearly polarized laser, and the disparity in photon populations between RCP and LCP light. Specifically, assuming that the gain is constant, the output power is determined by the difference between gain and loss. When the gain significantly exceeds the threshold, the impact of asymmetric loss between LCP and RCP, induced by the ACD thin film, becomes less significant, leading to a smaller DOCP. In contrast, when the gain is close to the threshold, asymmetric losses dominate the balance between RCP and LCP populations, enhancing the DOCP. In certain cases, only one polarization may reach the lasing condition (i.e., gain > loss), which can result in a perfectly circular polarization (DOCP = 1). This DOCP value is consistent with the expectation based on the results of the sum of single-pass front and back *g*_abs_ (∼−0.17 at a wavelength of 420 nm) and the cavity enhancement factor (Finesse/π ∼ 6).

It is worth noting that this differential absorption effect persists even below the lasing threshold (i.e., in the spontaneous emission regime), meaning the stimulated emission within the gain medium itself could not directly contribute to DOCP. Instead, the DOCP is predominantly induced by the thin film’s asymmetric absorption. The primary role of the stimulated emission in the gain medium is to generate linearly polarized light (with equal RCP and LCP photon numbers), while the DOCP is influenced by the DOP provided by the linearly polarized pump laser.

Due to the nonuniformity of the thin film, the single-pass CD varies at different points, which may influence the final DOCP. A preliminary CD map of the PTPO-coated mirror (shown in the Supporting Information) was measured before the mirror was placed into the laser cavity. We did not observe a significant impact of dye concentration or threshold energy on the DOCP. While the threshold energy varied with different dye concentrations and exposure times, the maximum DOCP consistently occurred near the threshold energy. These observations are in agreement with those of our model.

Additionally, we tested PTPO films of different thicknesses (300 and 100 nm), with their CD values measured using a Jasco J-1500 spectropolarimeter, yielding averaged CD values of 1000 mdeg and 170 mdeg, respectively. For the 100 nm film, the threshold was lower, but the DOCP remained below 0.15. In contrast, the 300 nm film, which resulted in greater absorption loss, had a higher threshold but exhibited a more pronounced DOCP (>0.3). These results are in line with our expectations.

## Conclusions

To explore the potential of using ACD from 2D chiral organic thin films for generating CP light, we developed a free-space dye laser system with a chiral cavity incorporating such a thin film. Our experimental results demonstrate the successful generation of CP laser light, achieving a DOCP as high as 0.6, along with a DOP approaching 0.8, indicating superior control over the polarization state. The ellipticity of the emitted light is attributed to the ACD effect of the 2D chiral thin film, which induces asymmetric cavity losses between the LCP and RCP light. We observed dynamic changes in the CP characteristics as a function of pump energy with DOCP increasing initially and then decreasing as pump power rises. This behavior is explained by using a simple laser pulse evolution model, shedding light on the mechanism behind the asymmetric loss contributions.

This work demonstrates the potential of integrating 2D chiral thin films into laser systems to directly generate CP light, paving the way for the development of organic, chiral light-emitting devices. Our results demonstrate that with precise control of the pump power in a continuous-wave laser, it is possible to selectively suppress one CP light while enabling the other to reach the lasing threshold. This suggests that the DOCP could potentially approach unity under the optimized conditions. While we implemented the thin film in a free-space laser system, its application is not limited to this setup and could extend to other existing laser technologies, such as microfluidic dye lasers,^38^ miniaturized or chip-scale systems. These findings underscore the versatility of ACD-based thin films for both fundamental research in chiral photonics and practical applications requiring controlled polarization states, opening new pathways for advancing chiral photonic technologies.

## Supplementary Material



## Notes

Details on the experimental procedures, including the fabrication of the PTPO chiral thin film, and describes the setup used to map the absorption differences between left- and right-circularly polarized light at various positions on the PTPO-coated mirror; validation of a custom-built polarimeter for Stokes vector measurements, with performance comparisons against commercial polarimeters; and the data analysis and binning processes, along with the use of the Qscope system for pulse signal acquisition and processing (PDF)

## Notes

L.-Z.L.; L.-Q.H.; S.-W.Y.; Y.-J.H.; F.Z.; A.S.; L.D.B.; R.H.G.; R.T.; C.-Y.H.; T.-L.C. Circularly Polarized Stimulated Emission from a Chiral Cavity Based on Apparent Circular Dichroism Organic Thin-Films. ChemRxiv. 2025; doi: 10.26434/chemrxiv-2025-2xsvw. This content is a preprint.
