# Supplementary material for: Circularly Polarized Stimulated Emission from a Chiral Cavity Based on Apparent Circular Dichroism Organic Thin Films
Source: ACS Photonics. 2025 Mar 19;12(5):2557–65. doi: 10.1021/acsphotonics.4c02560 (PMC12100771; doi:10.1021/acsphotonics.4c02560)
Supplement: Supplementary file 1 [file ph4c02560_si_001.pdf]

SUPPORTING INFORMATION FOR PUBLICATION

Circularly Polarized Stimulated Emission from a Chiral Cavity Based on Apparent Circular Dichroism Organic Thin-Films

Authors:
Li-Zhi Lin[1], Ling-Qi Huang[1], Shi-Wei You[1], Yi-Jan Huang[1], Francesco Zinna[2], Andrew Salij[3], Lorenzo Di Bari[5], Randall H. Goldsmith[4], Roel Tempelaar[3], Chia-Yen Huang[1], Tzu-Ling Chen[1,*]

Affiliations:
[1]Department of Photonics, National Yang-Ming Chiao-Tung University, Hsinchu, 300013, Taiwan
[2]2Dipartimento di Chimica e Chimica Industriale, Universit'a di Pisa, Pisa, PI, 56124, Italy
[3]Department of Chemistry, Northwestern University, Evanston, IL, 60208, USA
[4]Department of Chemistry, University of Wisconsin-Madison, Madison, WI, 53706, USA

*Corresponding author: tlc@nycu.edu.tw

1. MIRROR COATING

For this experiment, we chose a plane-concave cavity design, where the concave mirror had 99% reflectivity, and the planar mirror was a high-reflectivity coverslip coated with a chiral PTPO thin film. The plane mirror coating spec is illustrated in Figure S1.

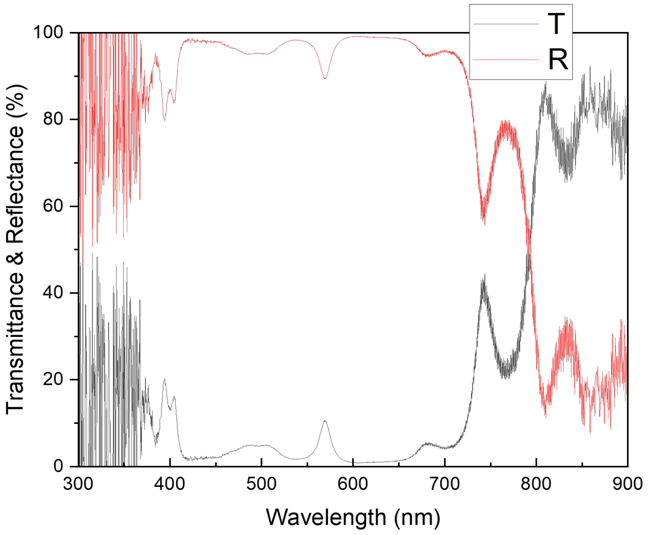

Fig. S1. Reflectance and transmittance of the HR coverslips mirror (the planar mirror in the laser cavity)

2. FABRICATION OF PTPO CHIRAL THIN FILM

The fabrication process of the PTPO thin film involved cleaning glass substrates with acetone at 40° C and isopropyl alcohol at 80° C, followed by air plasma treatment for surface activation. PTPO is dissolved in CH_2Cl_2 at a concentration of 4×10^{-2}M, then 100 μL of the solution is spin-coated onto a 25×25 mm^2 glass substrate at 2000 rpm for 30 seconds, with an acceleration of 1000 rpm/s. The film is then thermally annealed at 80°C for 1 hour, resulting in 10 films approximately 300 nm thick. After annealing, the films are analyzed using ECD spectroscopy.

Figure S2 shows the absorption profiles of the glasses coated with the film. The estimation for the dielectric tensor and extinction coefficients for PTPO can be found in Figures S1-S2 for the paper ChemRxiv. 2024; doi:10.26434/chemrxiv-2024-hj1fl, and corresponding raw data will be provided when that paper is published.

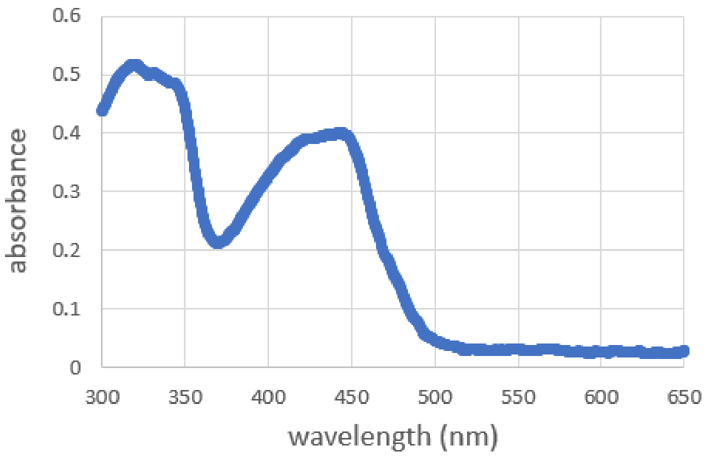

Fig. S2. The absorption profile of 300 nm thick PTPO films.

3. CHARACTERIZATION OF CD MAP

Because the thin film is not uniform, performance varies at different points. To identify optimal regions, we first obtained a preliminary CD map of the PTPO-coated mirror before placing it into the laser cavity. As shown in S3(a), a white light source with a polarization beam splitter (PBS) and a quarter-wave plate (QWP) produces circularly polarized light. Rotating the QWP to 45° generates left-handed circular polarization (LCP), and at 135° generates right-handed circular polarization (RCP). A spectrometer behind the sample measures the absorption differences between RCP and LCP at various positions, allowing calculation of the CD distribution on the mirror's surface.

Figure S3(b) shows the resulting CD map. The absorption differences translate into variations in emission intensity, quantified by the "Difference" value defined below. Points with higher CD performance can then be selected for subsequent measurements.

$$A_{\mathrm{LCP}} = -\log_{10}\left(\frac{I_{\mathrm{LCP}}}{I_0}\right) \qquad A_{\mathrm{RCP}} = -\log_{10}\left(\frac{I_{\mathrm{RCP}}}{I_0}\right)$$

$$\Delta A = A_{\mathrm{LCP}} - A_{\mathrm{RCP}} \qquad \mathrm{Difference} = \left|\frac{2 \times (\mathrm{Intensity}_{\mathrm{LCP}} - \mathrm{Intensity}_{\mathrm{RCP}})}{\mathrm{Intensity}_{\mathrm{LCP}} + \mathrm{Intensity}_{\mathrm{RCP}}}\right|$$

4. DEVELOPMENT AND VALIDATION OF A HOME-BUILT POLARIMETER FOR STOKES VECTOR MEASUREMENT

To characterize the polarization state of light, we used Stokes vectors. Due to the low repetition rate (10 Hz) of our pulsed laser, most commercial polarimeters are unsuitable, so we developed a custom system incorporating a QWP and polarizer, with the QWP mounted on an electronically controlled stage (Sigmakoki, GSC-01) to reduce manual adjustment errors. We measured eight QWP angles to construct a complete Stokes vector. To validate our system, we compared its measurements with those from a commercial polarimeter using a 405 nm CW laser, under various polarization states (LCP, RCP, linear, and random), generated with an additional QWP (Thorlabs, AQWP05M-600) and PBS (Thorlabs, PBS201).

The reliability of our system was assessed by comparing its linearity and Stokes vector results with those from a commercial polarimeter and oscilloscope. The linearity comparison is shown in Figure S5. Discrepancies were found to be within 10%.

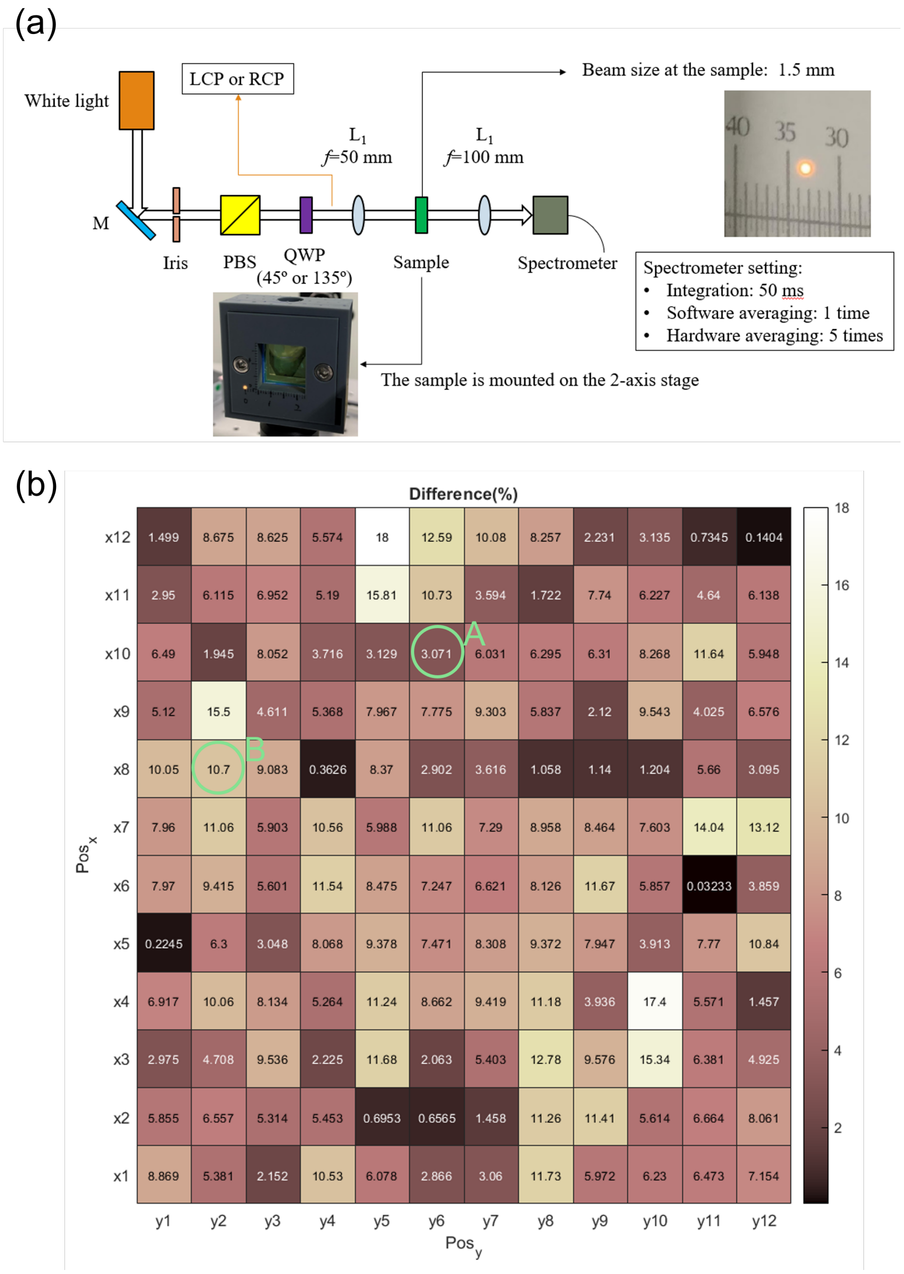

Fig. S3. (a) Experimental setup for characterizing CD of the PTPO coated mirror. PBS and QWP were placed in front of the sample. By adjusting the QWP angle to 45° and 135°, LCP and RCP light were produced, respectively. The intensities of these two polarized lights were subsequently measured using a spectrometer. (b) Map showing the absolute values of the subtraction difference between LCP and RCP absorption across different regions of a chiral material mirror. The mirror has dimensions of 18 mm × 18 mm, divided into a 12 × 12 grid of square regions, each measuring 1.5 mm. The horizontal axis represents the y-position and the vertical axis represents the x-position of each grid cell. The color scale indicates the magnitude of the difference in absorption between left- and right-circularly polarized light.

5. DATA ANALYSIS AND BINNING

The Qscope is a custom software system developed in our laboratory, based on Field Programmable Gate Array (FPGA) programming. It utilizes the Zynq UltraScale+™ MPSoC architec-

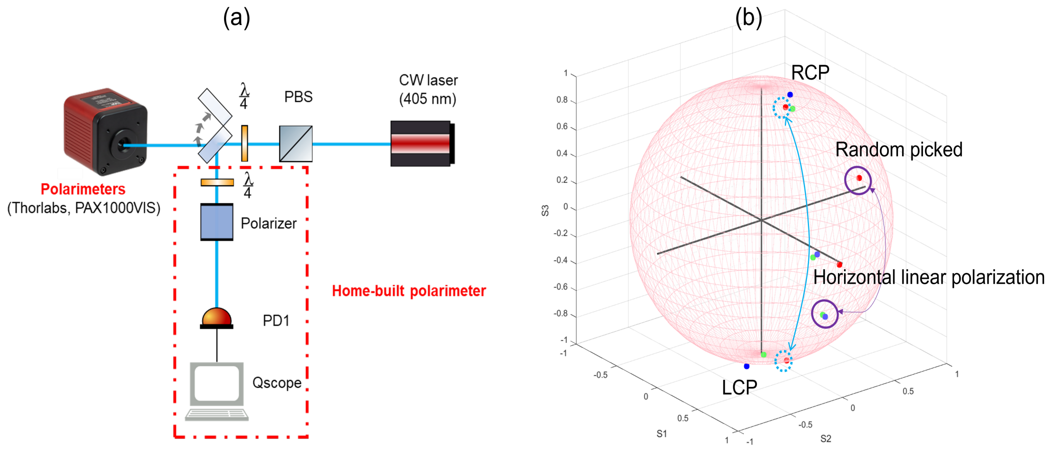

Fig. S4. (a) The experimental setup for verifying the stokes vector measurements between the commercial instrument (Thorlabs, PAX1000VIS) and the home-built polarimeter. (b) Comparison of Results from Commercial Polarimeter (Red), Oscilloscope (Green), and Qscope (Blue) on the Poincaré Sphere. The results demonstrate consistency between the commercial and home-built polarimeters for the same polarization states. Notably, the results exhibit an opposite sign difference for S3 value, as the light passing through the home-built polarimeter is reflected from an additional mirror, which reverses the direction of circular polarization rotation.

ture, which combines both the processing system and programmable logic sections. Operating similarly to an oscilloscope, the Qscope provides two input channels, two output channels, and a built-in function generator capable of producing various signal waveforms. Additionally, it supports external trigger inputs. While the Qscope has a sampling rate of 250 MS/s, its significantly higher frame rate enables more efficient data acquisition compared to traditional oscilloscopes, making it particularly suitable for this experiment.

Figure S6(a) shows a typical pulse signal recorded by the Qscope, with the y-axis representing voltage (in Volts) and the x-axis corresponding to time (in units of the inverse of the Qscope sampling rate). The plot captures the intensity variation of the laser output over time, displaying a prominent peak in the center, reflecting the maximum output of the laser pulse.

The maximum value of each laser pulse was extracted and recorded as the peak for that pulse. For each QWP rotation angle, measurements were taken over a 3-minute period, during which the pulse energy fluctuated significantly. Under a fixed pump power, all pulse values were accumulated in a single plot, as shown in the top left panel of Figure S6(b), where PD1 and PD2 recorded pulse peaks for the pump and dye laser outputs, respectively. The data was then grouped by energy levels, and a binning plot was generated, shown in the top right panel of Figure S6(c).

After binning, the relationship between pump energy and dye laser output energy was plotted for all 9 angles, as shown in Figure S6(d). The QWP at 180° should be identical to the 0° position in physical conditions, which was used to confirm the system's consistency during the entire measurement. These angles were used to calculate the final Stokes vectors.

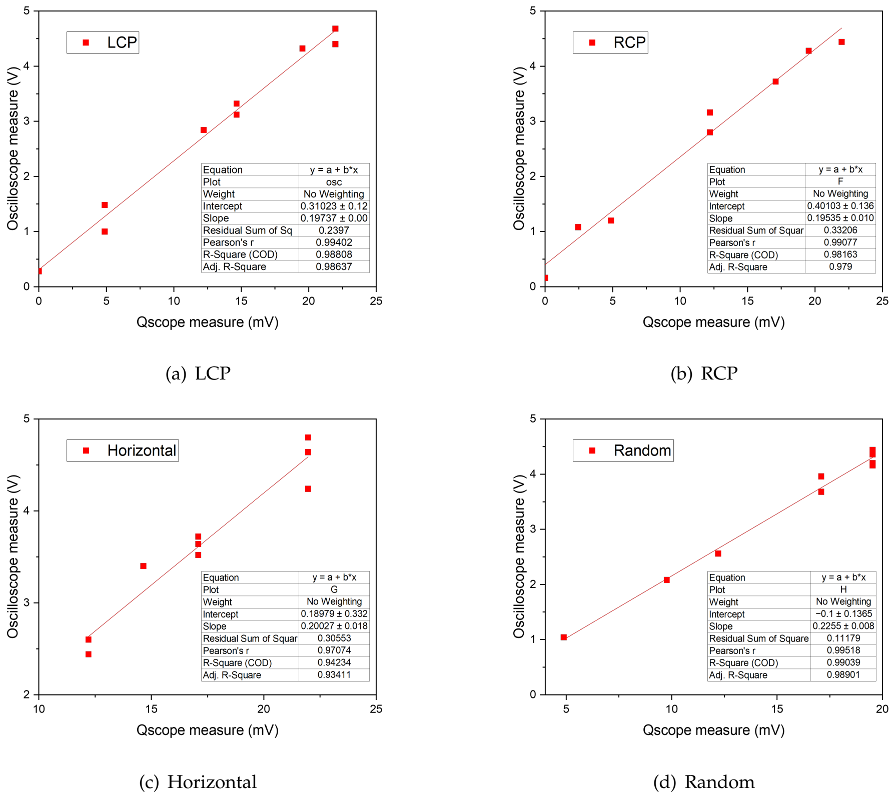

(a) LCP

(b) RCP

(c) Horizontal

(d) Random

Fig. S5. Comparison between Qscope and general oscilloscopes

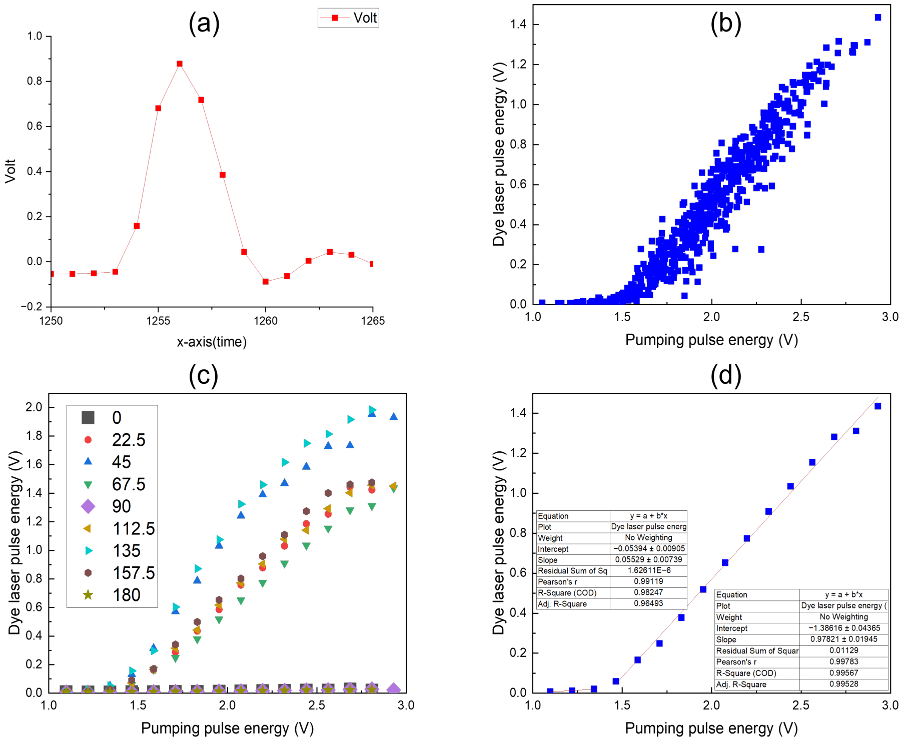

Fig. S6. (a) Laser pulse signal profile recorded by Qscope, where the time axis is in units of the Qscope sampling rate (around 4 ns). (b) Pump energy vs. dye Laser output energy for 9 QWP rotation angles: (b) Scatter plots before binning, (d) after binning, and (c) overall angular dependence, which contributes to the calculation of the final Stokes vector.
